# Editorial: Immunotherapy and cell therapy for patients with relapsed and refractory acute myeloid leukemia

**DOI:** 10.3389/fonc.2025.1691911

**Published:** 2025-10-27

**Authors:** JingYi Huang, ShiYao Song, Xiaotian Zhang, Chunliang Li, Yan-Lai Tang

**Affiliations:** ^1^ Department of Pediatrics, First Affiliated Hospital of Sun Yat-sen University, Guangzhou, China; ^2^ Department of Biochemistry and Molecular Biology, University of Texas Health Science Center Houston, Houston, TX, United States; ^3^ Department of Tumor Cell Biology,St Jude Children’s Research Hospital, Memphis, TN, United States

**Keywords:** AML, immunotherapy, CAR-T, cell therapy, cardiac tamponade

The treatment of patients with relapsed or refractory AML (R/R AML) remains a major challenge attributed to limited treatment measures and the lack of standardized treatment protocol. The advent of immunotherapy and cell therapy, particularly chimeric antigen receptor T-cell (CAR-T) therapy and immune checkpoint inhibitors, has opened new avenues for improving treatment outcomes in these patients.

One promising approach is the use of immunotherapy and cell therapy for treating R/R AML (Bawek et al.). The review highlights the potential of immune checkpoint inhibitors, particularly PD-1/PD-L1 blockers, when combined with chemotherapy and hypomethylating agents. CAR-T cell therapy, targeting specific antigens like CD7 and CD33, shows promising results, but challenges remain regarding target selection and managing side effects. The review also emphasizes the importance of understanding the immune microenvironment and personalized treatment strategies to improve outcomes in patients who are not eligible for stem cell transplants.

In addition, CAR-T cell therapy has demonstrated significant promise in treating R/R ALL. Liu et al. reports one successful treatment of a patient with R/R B-cell ALL involving both bone marrow and central nervous system using sequential autologous and allogeneic CD19-targeted CAR-T therapies. Initially, the autologous CAR-T treatment achieved complete remission in both the bone marrow and cerebrospinal fluid for 40 days, but relapse occurred in the bone marrow. Allogeneic CAR-T therapy was subsequently administered, leading to another complete remission. Despite mild cytokine release syndrome and neurotoxicity, the treatment was effective, bridging the patient to hematopoietic stem cell transplantation with five months of disease-free survival.

Another case report also showed the promising future of CAR-T therapy. Han et al. successfully treated a patient with mixed-phenotype acute leukemia (MPAL) who relapsed after allogeneic hematopoietic stem cell transplantation (allo-HSCT). The treatment led to negative measurable residual disease (MRD) in both the myeloid and T-lineages. The patient achieved complete remission (CR) and remained MRD-negative for four months post-treatment. This case demonstrates that CD7-targeted CAR-T therapy, combined with demethylating agents, offers a promising treatment option for MPAL relapsed after allo-HSCT.

Additionally, venetoclax, when used in combination with hypomethylating agents and the CAG regimen (Dac + ACR + Ara-C + G-CSF), has shown encouraging results in R/R AML patients. In a trial by Liu et al., 90% of patients responded to the VEN-DCAG regimen, with 85% achieving CR or CRi. MRD-negative remission was observed in 76.5% of patients, and the regimen showed high efficacy in both relapse and refractory groups. These outcomes are particularly notable given the high-risk nature of R/R AML patients, demonstrating the potential of combining targeted therapies with epigenetic modulators to enhance remission rates.

Beyond immunotherapy, R/R AML patients may also experience severe complications such as cardiac tamponade, a rare and life-threatening condition. Li et al. highlighted the clinical features, diagnostic approach, and treatment outcomes of a case series. 5 pediatric AML patients initially presented with cardiac tamponade, with 2 surviving after aggressive chemotherapy and pericardial drainage, while 3 patients succumbed within two weeks. This article alarms us of the importance of early diagnosis and timely management to reduce mortality, along with the challenges of treating AML with cardiac myeloid sarcoma.

Immunotherapy and cell therapy have brought new hope for the treatment of patients with R/R AML. For instance, Liu et al. achieved a very good response rate in the treatment of patients with recurrent T-ALL and MPAL using CAR-T cells targeting CD7. Han et al. reported a successful case of combined treatment with CD7-targeted CAR-T cells and demethylating drugs, in which the patient achieved complete remission after recurrence after transplantation. The combination of targeted therapies like Venetoclax and demethylating agents has improved remission rates, especially in high-risk patients (Liu et al.). However, complications such as cardiac tamponade in AML patients further complicate treatment, emphasizing the need for comprehensive care strategies (Li et al.). Despite these challenges, these emerging therapies provide promising treatment options for AML and MPAL patients, requiring continued research to refine treatment regimens and improve long-term survival.

